# Comparative transcriptome meta-analysis reveals a set of genes involved in the responses to multiple pathogens in maize

**DOI:** 10.3389/fpls.2022.971371

**Published:** 2022-09-15

**Authors:** Yapeng Wang, Ting Li, Zedan Sun, Xiaojian Huang, Naibing Yu, Huanhuan Tai, Qin Yang

**Affiliations:** ^1^State Key Laboratory of Crop Stress Biology for Arid Areas and College of Agronomy, Northwest A&F University, Yangling, China; ^2^Key Laboratory of Maize Biology and Genetic Breeding in Arid Area of Northwest Region of the Ministry of Agriculture, Northwest A&F University, Yangling, China

**Keywords:** maize, pathogens, meta-analysis, multiple disease resistance, differentially expressed genes (DEGs)

## Abstract

Maize production is constantly threatened by the presence of different fungal pathogens worldwide. Genetic resistance is the most favorable approach to reducing yield losses resulted from fungal diseases. The molecular mechanism underlying disease resistance in maize remains largely unknown. The objective of this study was to identify key genes/pathways that are consistently associated with multiple fungal pathogen infections in maize. Here, we conducted a meta-analysis of gene expression profiles from seven publicly available RNA-seq datasets of different fungal pathogen infections in maize. We identified 267 common differentially expressed genes (co-DEGs) in the four maize leaf infection experiments and 115 co-DEGs in all the seven experiments. Functional enrichment analysis showed that the co-DEGs were mainly involved in the biosynthesis of diterpenoid and phenylpropanoid. Further investigation revealed a set of genes associated with terpenoid phytoalexin and lignin biosynthesis, as well as potential pattern recognition receptors and nutrient transporter genes, which were consistently up-regulated after inoculation with different pathogens. In addition, we constructed a weighted gene co-expression network and identified several hub genes encoding transcription factors and protein kinases. Our results provide valuable insights into the pathways and genes influenced by different fungal pathogens, which might facilitate mining multiple disease resistance genes in maize.

## Introduction

Plants live in a challenging environment hosting a wide array of microbial pathogens, including viruses, bacteria, fungi, and oomycetes. An increasing number of infectious crop diseases reduce crop yields greatly and threaten global food security. To fight off biological threats, plants have evolved a two-layered defense system (Jones and Dangl, 2006; Zhou and Zhang, 2020; Wang Y. et al., 2022). In the first layer, pattern recognition receptors (PRRs) on the cell surface recognize conserved microbial features called pathogen-associated molecular patterns (PAMPs), leading to PAMP-triggered immunity (PTI) (Couto and Zipfel, 2016; Gust et al., 2017). Adapted pathogens have evolved effectors to overcome PTI. In the second layer, pathogen effectors are directly or indirectly recognized by intracellular nucleotide-binding leucine-rich repeat receptors (NLRs), activating effector-triggered immunity (ETI) (Lolle et al., 2020). PTI and ETI lead to a suite of shared downstream responses, such as calcium influx, reactive oxygen species (ROS) burst, phytohormone modulation, and biosynthesis of a diverse set of secondary metabolites (Peng et al., 2018). Recent studies showed that PTI and ETI reinforce each other to confer more robust defense responses against pathogens (Ngou et al., 2021; Yuan et al., 2021).

Receptor-like kinases (RLKs), and receptor-like proteins (RLPs) are the major cell surface localized PRRs. PRRs often lead to partial disease resistance or quantitative disease resistance, which has been widely used in crop breeding and tends to confer durable and multiple disease resistance (Kanyuka and Rudd, 2019). Based on their extracellular domain, PRRs were divided into leucine-rich repeat (LRR) RLKs, lysin motif (LysM) RLKs, lectin RLKs, and wall-associated kinase (WAK) subfamilies (Tang et al., 2017). A significant number of LRR-RLKs or LRR-RLPs have been characterized to be involved in disease resistance in Arabidopsis, rice, and tomato (Song et al., 1995; Dixon et al., 1996; Chinchilla et al., 2007; Boutrot and Zipfel, 2017). The most well-described LRR-RLK is FLS2 in Arabidopsis, which directly binds with bacterial flagellin and forms a signaling complex with its co-receptor BAK1 to activate downstream defense responses (Chinchilla et al., 2007). The rice *Xa21* gene confers resistance to multiple races of bacterial blight tested (Song et al., 1995; Wang et al., 1996). In maize, very few LRR-RLKs have been reported that might be associated with disease resistance, including pan1 and FI-RLPK (Jamann et al., 2014; Block et al., 2021). The WAK-RLKs have been reported as major players in disease resistance of cereal crops, such as ZmWAK against maize head smut (Zuo et al., 2015), ZmWAK-RLK1 against northern leaf blight (NLB) in maize (Hurni et al., 2015), and Stb6 against septoria tritici blotch disease in wheat (Saintenac et al., 2018). Typical WAK-RLK protein includes an extracellular galacturonan-binding (GUB) domain, an epidermal growth factor (EGF)-like domain, a transmembrane domain, and an intracellular kinase domain. WAK-RLKs have been reported to recognize cell-wall derived molecules, such as oligogalacturonides (OGs), and transmit signals to the cytoplasm to initiate defense responses (Kanyuka and Rudd, 2019).

Plants also produce diverse secondary metabolites to protect against pathogen attack, such as terpenoids and lignin. Terpenoid phytoalexins zealexins and kauralexins have been recognized as significant contributors to multiple pathogens response in maize (Ding et al., 2019). Lignin, which is synthesized through the phenylpropanoid pathway, has been reported to play a critical role in providing partial resistance to one or more pathogens (Dong and Lin, 2021). Activation of rice *4-coumarate:coenzyme A ligase* genes (*Os4CL3* and *Os4CL5*) by OsMYB30 resulting in accumulation of lignin subunits G and S and inhibiting *Magnaporthe oryzae* penetration at the early stage of infection (Li et al., 2020). *ZmCCoAOMT2*, encoding a caffeoyl-CoA O-methyltransferase, contributes to multiple disease resistance through the phenylpropanoid pathway and lignin accumulation (Yang et al., 2017c). Moreover, natural variation of a maize F-box gene (*ZmFBL41*) results in the inhibition of ZmCAD degradation and accumulation of lignin, leading to enhanced banded leaf and sheath blight resistance (Li N. et al., 2019).

Nutrient access is arguably the most limiting factor during pathogens invasion. An increasing number of studies have shown that nutrient transporter genes play important roles in plant disease resistance (Chen et al., 2010; Moore et al., 2015; Sonawala et al., 2018; Eom et al., 2019). Pathogens may reprogram host plants’ nutrient metabolism to facilitate their growth and invasion. Sugar transporters named SWEETs are hijacked by pathogens for the supply of sucrose to achieve successful colonization and infection (Eom et al., 2019). Sugar transport proteins (STPs), which belong to a large subfamily of the monosaccharide transporter, have been reported to function in defense response (Yamada et al., 2016; Liu et al., 2021). The wheat *Lr67* gene encoding a hexose transporter confers adult plant resistance to multiple wheat rust pathogens (Moore et al., 2015). Amino acid transporters (AATs) are membrane-bound transporter proteins that mediate the transfer of amino acids in or out of plant cells, which have been shown to contribute to susceptibility in plants (Elashry et al., 2013; Berg et al., 2021; Tünnermann et al., 2022). In cucumber and tomato, *CsAAP2A* and *SlAAP5A/B* have been identified as susceptibility genes for oomycete pathogens (Berg et al., 2021).

Maize (*Zea mays L.*) is one of the most widely cultivated crops, consistently plagued by a variety of fungal diseases, such as southern leaf blight, northern leaf blight, gray leaf spot, stalk rot, and ear rot (Yang et al., 2017b). To defend against pathogens invasion, maize has evolved a complex array of defense strategies. Hundreds of disease resistant quantitative trait loci (QTL) have been reported in maize, but few genes have been cloned and validated (Zhao et al., 2015; Rossi et al., 2019). The molecular mechanisms of multiple disease resistance are largely unknown. Over the years, large transcriptome profiling studies have been used to reveal how maize lines responded to different pathogens infections, while these studies have mostly focused on a single pathogen. In this study, we chose publicly available RNA-seq data sets from seven independent maize pathogen inoculation experiments to investigate the molecular mechanisms underlying maize response to different pathogen infections. A set of key pathogen-responsive pathways and genes were identified using the integrated bioinformatics pipeline. The results of this study provide valuable insights into the pathways and genes influenced by different pathogens, which would facilitate mining multiple disease resistance genes in maize.

## Materials and methods

### RNA-seq data sets, mapping, and differential gene expression analyses

FASTQ files from six published RNA-seq studies were downloaded from the European Nucleotide Archive database (Supplementary Table 1). Raw reads were trimmed by fastp and reads quality was assessed by FastQC. All cleaned reads were mapped to the B73 reference genome (RefGen_v5) using Hisat2-2.0.4 with default parameters (Kim et al., 2015). SAMtools was used to sort and convert SAM files to BAM files. StringTie was used to assemble the BAM file and measure the expression levels of genes (Pertea et al., 2016). Transcripts per million (TPM) values of all data sets were extracted using StringTie. For differentially expressed genes (DEGs) analysis, a python script prepDE was utilized to get genes count matrices. DEGs were determined with a stringent criterion [| log2(fold change)| ≥ 1, *p.adj* < 0.05] using the R package DESeq2.

### Exploratory data analysis

Principal component analysis (PCA) was performed to estimate the biological replications of each sample. The common DEGs (co-DEGs) of the four maize leaf inoculation experiments and all the seven inoculation experiments were determined using the R package UpSetR (Conway et al., 2017). Kauralexin and lignin biosynthesis-related genes were selected as reported previously (Supplementary Table 2) (Yang et al., 2017a; Ding et al., 2019). For nutrient transporters analysis, we downloaded PF00083, PF03083, and PF01490 HMM profiles from Pfam database, using HMMER software with default parameters to search for sugar transporters, sweet transporters, and amino acid transporters (Mistry et al., 2013). For potential PRRs analysis, hmmscan was used to find conserved protein domains with default parameters. The selected sequences were then analyzed with HMMER^1^ to assure the presence of the membrane-spanning domain, and the kinase domain.

### Gene set enrichment and protein functional analysis

Gene ontology (GO) term enrichment of gene set was performed using the R package clusterprofiler with a cut-off of *p* < 0.01. GO terms were annotated by eggnog-mapper software (Huerta-Cepas et al., 2017). Kyoto Encyclopedia of Genes and Genomes (KEGG) pathways enrichment was performed by KOBAS v3.0 software^2^ based on the Benjamini and Hochberg false discovery rate correction. KEGG pathways with a corrected *p*-value < 0.05 were defined as significantly enriched (Xie et al., 2011). PlantTFDB database^3^ was used to determine transcription factors (TFs) base on amino acid sequence similarity (Jin et al., 2017). Hmmscan was used to find protein kinases (PKs) with default parameters.

### Co-expression network analysis

To reveal potential pathogen-responsive modules, the R package WGCNA was used to construct gene co-expression network (Langfelder and Horvath, 2008). Normalized TPM matrix of all the samples was obtained by a python script. An unsigned type of weighted gene co-expression network analysis (WGCNA) network was created using the following parameters: the soft-threshold value was set to 13, the threshold for merging of modules was set to 0.25 and the minimum module size was set to 30. WGCNA edge weight (ranging from 0 to 1) > 0.1 was exported and visualized using Cytoscape 3.8.2 (Shannon et al., 2003).

### Quantitative reverse transcription PCR validation of representative candidate genes

Maize inbred line B73 was inoculated with *Cochliobolus heterostrophus* on leaves, *Fusarium graminearum* on roots, and *Fusarium verticillioides* on ears, respectively. For *C. heterostrophus* inoculation experiment, maize inbred line B73 was grown at 26 ± 1°C with 16 h of light and 8 h of darkness. The fourth leaves were spray-inoculated with *C. heterostrophus* spores suspension (5 × 10^4^ml^–1^) in 0.05% agar and a 0.05% Tween 20 as described before (Belcher et al., 2012). Leaf samples were collected at 0, 2, and 6 hpi. Each sample was pooled from three plants. For *F. graminearum* inoculation experiment, B73 was germinated at 26 ± 1°C with 16 h of light and 8 h of darkness until roots were 6–8 cm long. The primary roots were soaked in the *F. graminearum* spore suspension (6 × 10^6^ml^–1^) in 3% liquid mung bean broth and incubated at 26°C with 50 rpm rotation (Ye et al., 2013). The inoculated seedling roots were harvested at 0, 2, and 18 hpi. Each sample was pooled with six plants. For *F. verticillioides* inoculation experiment, B73 was grown in the field and the ears were harvested 15 days after pollination. Each kernel was cut in the middle, with one half being soaked in sterile distilled water and the other half being soaked in the suspension of *F. verticillioides* spores (5 × 10^6^ml^–1^), and incubated for 1 h at 26°C with 50 rpm rotation as reported before (Yao et al., 2020). The kernel halves soaked in distilled water were sampled at 0 hpi. Thereafter, all kernel halves were transferred to potato dextrose agar medium and cultured at 28°C. The inoculated ears were harvested at 0, 2, and 6 hpi. Each sample was pooled with nine kernels from three ears. In each of the experiments, 0 hpi represents before inoculation. We conducted two biological replicates for each experiment and all samples were frozen in liquid nitrogen immediately and stored at -80^°^C until RNA extraction.

Total RNA from all samples was extracted using Plant RNA Kit (Omega Bio-tek, United States). The quality and concentration of RNA were determined with a Nanodrop spectrophotometer. The cDNA was synthesized using the FastKing RT Kit (Tiangen, China). Quantitative reverse transcription-PCR (qRT-PCR) was performed on a QuantStudio 7 Flex Real-Time PCR System (Thermo Fisher, America) with a SuperReal PreMix Plus kit (SYBRGreen) (Tiangen, China). Primer sequences of representative co-DEGs were designed using Primer 5.0 and listed in Supplementary Table 3. The maize gene *ZmEF-1*α was used as an internal reference gene to normalize the relative expression of candidate genes. Each expression analysis was carried out for two biological replicates with three technical replicates for each biological replicate. The relative expression level of each gene was calculated using the 2^–ΔΔCt^ method.

## Results

### Source and overview of RNA-seq datasets

**FIGURE 1:**
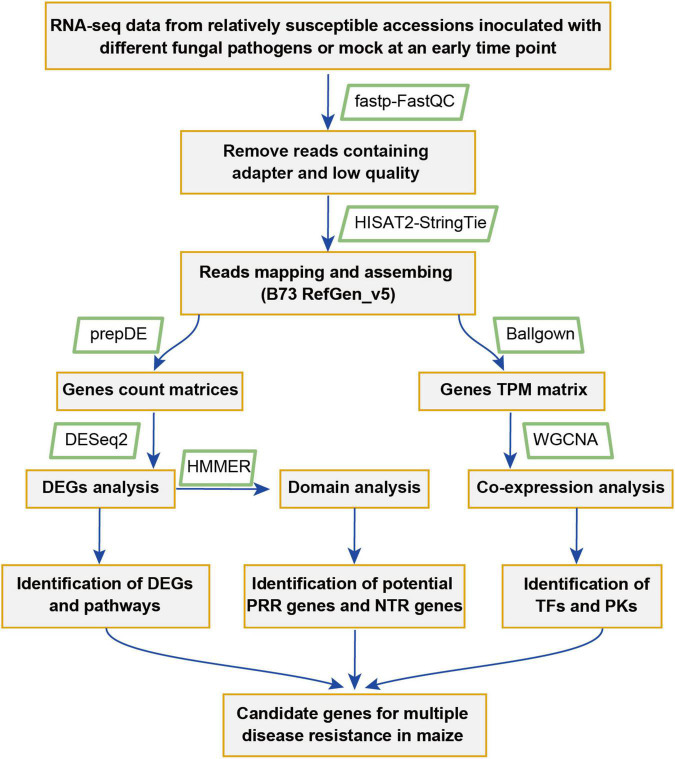
A flowchart showing the meta-analysis procedure used for the identification of genes and pathways putatively involved in multiple pathogens responses in maize.

To identify key genes that might play important roles in maize disease resistance, a comparative transcriptomic meta-analysis was performed using seven published RNA-seq datasets. Four datasets were generated from maize leaves inoculated with *Cochliobolus heterostrophus* (causal agent of southern leaf blight) (Ding et al., 2019), *Exserohilum turcicum* (causal agent of northern leaf blight) (Yang et al., 2019), *Cercospora zeina* (causal agent of gray leaf spot) (Swart et al., 2017), or *Colletotrichum graminicola* (causal agent of anthracnose leaf blight) (Hoopes et al., 2019). The other three datasets include maize root samples infected with *Fusarium graminearum* (causal agent of Gibberella stalk rot) (Liu et al., 2016) and maize kernels inoculated with *Aspergillus flavus* (causal agent of Aspergillus ear rot) or *Fusarium verticillioides* (causal agent of Fusarium ear rot) (Shu et al., 2017) (Table 1). We chose 34 RNA-seq data generated from relatively susceptible accessions inoculated with different fungal pathogens or mock (Supplementary Table 1). The detailed information of the RNA-seq data used in this study is summarized in Table 1. The overall workflow from data mining to candidate genes identification is illustrated in Figure 1.

**TABLE 1:** Main features of RNA-seq datasets used for meta-analysis.

Name	Pathogens	Biological replicates	Library layout	Inoculation time	BioProject accession	References
*C.h*_group	*C. heterostrophus*	4	single-end	24hpi	PRJNA491716	Ding et al., 2019
*E.t*_group	*E. turcicum*	3	single-end	72hpi	PRJNA392457	Yang et al., 2019
*C.z*_group	*C. zeina*	3	paired-end	Field	PRJNA369690	Swart et al., 2017
*C.g*_group	*C. graminicola*	2	paired-end	24hpi	PRJEB10574	Hoopes et al., 2019
*F.g*_group	*F. graminearum*	2	paired-end	18hpi	PRJNA308408	Liu et al., 2016
*A.f*_group	*A. flavus*	2	single-end	72hpi	PRJNA418364	Shu et al., 2017
*F.v*_group	*F. verticillioides*	2	single-end	72hpi	PRJNA418364	Shu et al., 2017

### RNA-seq data analyses

**FIGURE 2:**
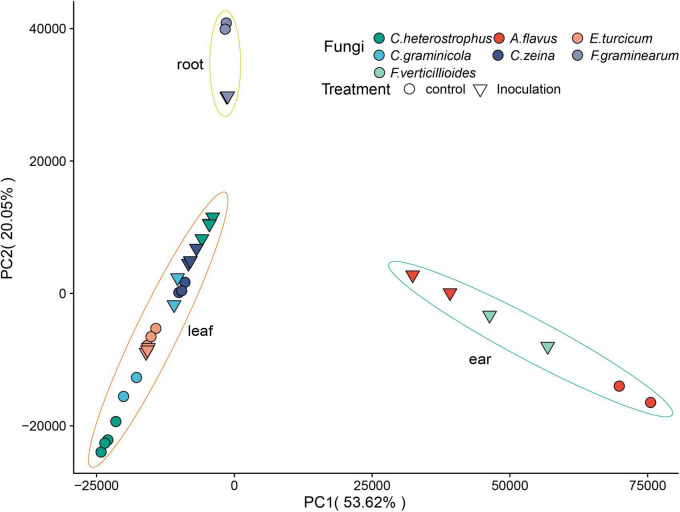
Principal component analysis of RNA-seq data. The treatment conditions and fungi are indicated by different symbols/colors in the plot.

A total of 1.7 billion high-quality reads were obtained after removing low-quality reads, and then mapped to the maize B73 reference genome (B73_RefGen_v5^4^). Sample relationships were estimated using principal component analysis (PCA). A very clear separation among tissues was observed (Figure 2). The replicates of each sample clustered together based on the first principal component (PC1) and the second principal component (PC2), suggesting the high reproducibility of the dataset (Figure 2). We normalized gene expression level as transcripts per million (TPM). To reduce the influence of lowly expressed genes, we filtered the gene if its average TPM value was ≤ 1. In total, 25,646 genes were detected across all the samples (Supplementary Table 4).

**FIGURE 3:**
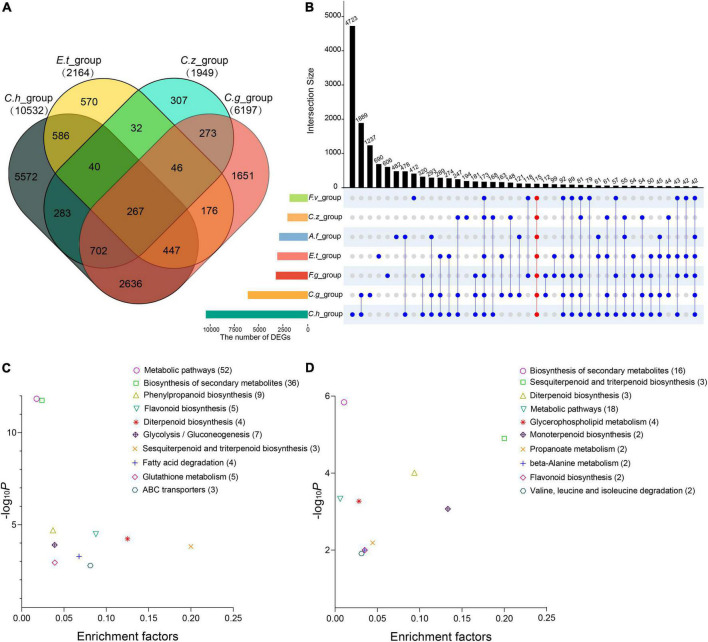
Differentially expressed genes (DEGs) in the four maize leaf infection experiments and in all the seven infection experiments. **(A)** Venn diagram showing the overlap of DEGs among the four maize leaf infection experiments. **(B)** UpSet diagram showing the overlap of DEGs among all the seven infection experiments. **(C)** Kyoto Encyclopedia of Genes and Genomes (KEGG) analysis of the co-DEGs among the four maize leaf infection experiments. **(D)** KEGG analysis of the co-DEGs among all the seven infection experiments. Abscissae represents the enrichment factor of each pathway and ordinate represents -log10 (P-value). Values in parentheses represent the number of components in each pathway.

Pairwise contrasts between the pathogen- and control-treated samples in each experiment were applied to identify differentially expressed genes (DEGs). As a result, we found 10,532, 2,164, 1,949, 6,197, 2,867, 2,957, and 1,875 DEGs in *C. heterostrophus* inoculation group (*C.h_group*), *E. turcicum* inoculation group (*E.t_group*), *C. zeina* inoculation group (*C.z_group*), *C. graminicola* inoculation group (*C.g_group*), *F. graminearum* inoculation group (*F.g_group*), *A. flavus* inoculation group (*A.f_group*), and *F. verticillioides* inoculation group (*F.v_group*), respectively (Supplementary Table 5). Among them, 267 common DEGs (co-DEGs) were determined in the four maize leaf inoculation groups (Figure 3A and Supplementary Table 6), and 115 co-DEGs were determined in all the seven inoculation groups (Figure 3B and Supplementary Table 6). It is worthy to mention that *C.h_group* had much more DEGs than the other groups, suggesting that maize plants might respond to *C. heterostrophus* very fast and 24 hpi is a relatively late time point.

### Dynamic transcriptome in maize response to multiple pathogens invasion

Plant immunity requires large-scale transcriptional reprogramming for proper immune output. In this study, we focused on the DEGs identified in the seven inoculation groups to view dynamic transcriptome responses for different pathogens. Gene Ontology (GO) enrichment and Kyoto Encyclopedia of Genes and Genomes (KEGG) pathways analyses were performed to annotate the DEGs from each group. As expected, GO terms related to plant-pathogen interactions were significantly enriched (*p* < 0.01), including photosynthesis, response to hydrogen peroxide, hormone metabolic process, secondary metabolic process, response to chitin, response to calcium ion, and carbohydrate derivative transport (Supplementary Table 7). We hypothesized that maize may employ similar defense mechanisms to combat different pathogens. To test the hypothesis, we selected 267 co-DEGs from the four maize leaf inoculation groups and 115 co-DEGs from all the inoculation groups to perform KEGG pathways analyses separately. The KEGG pathways that were most strikingly enriched among the co-DEGs included biosynthesis of secondary metabolites, diterpenoid biosynthesis, and phenylpropanoid biosynthesis (Figures 3C,D and Supplementary Figures 1, 2).

### Potential pattern recognition receptors in maize response to multiple pathogens invasion

To investigate potential *PRR* genes in maize response to multiple pathogens invasion, we scanned all the DEGs with the plant PRR domains by using hmmsan (Mistry et al., 2013). As a result, 115 *LRR-RLKs*, 3 *LysM-RLKs*, 34 *lectin-RLKs*, 34 *WAK-RLKs*, 13 *LRR-RLPs*, 2 *LysM-RLPs*, 1 *lectin-RLP*, and 1 *WAK-RLP* were identified from all the DEGs. Differential expression analysis showed that most of the *RLKs* and *RLPs* were up-regulated after pathogens infection. Among them, 63 *LRR-RLKs*, 2 *LysM-RLKs*, 23 *Lectin-RLKs*, 27 *WAK-RLKs*, 9 *LRR-RLPs*, and 2 *LysM-RLPs* were found to respond to at least two pathogens (Supplementary Table 8), including *ZmWAK-RLK1* (*Zm00001eb360640*), which has been reported as a quantitative disease resistance gene against northern leaf blight caused by *E. turcicum* (Hurni et al., 2015; Yang et al., 2019). Notably, two *LRR-RLKs* (*Zm00001eb170460* and *Zm00001eb293660*), two *Lectin-RLKs* (*Zm00001eb058940* and *Zm00001eb325300*) and five *WAK-RLKs* (*Zm00001eb124900*, *Zm00001eb126150*, *Zm00001eb156230*, *Zm00001eb177830*, and *Zm00001eb334620*) were found to be up-regulated by all the seven pathogens inoculation. In addition, we found that the majority of the identified *RLKs* and *RLPs* showed similar expression patterns for different pathogens infections (up-regulated or down-regulated).

### Phenylpropanoid and diterpenoid biosynthesis genes in response to multiple pathogens invasion

**FIGURE 4:**
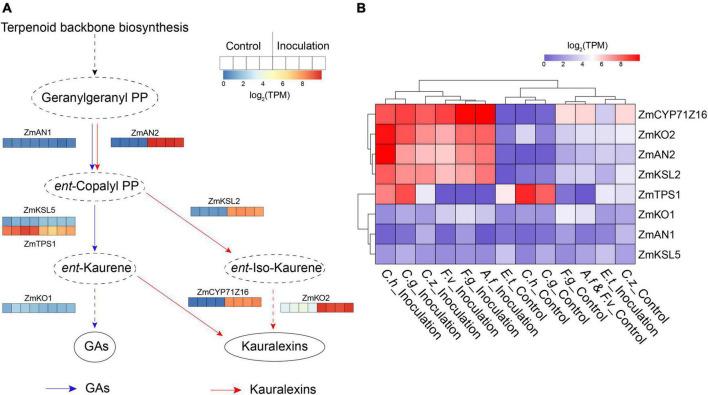
Expression patterns of kauralexin and gibberellic acid (GA) biosynthesis related genes. **(A)** Heat map of expression of kauralexin and GA biosynthesis related genes after inoculation with *Cochliobolus heterostrophus*. For each gene, the transcripts per million (TPM) value of four biological replicates is shown. **(B)** Heat map of expression of kauralexin and GA biosynthesis related genes after inoculation with all the seven independent pathogens. For each gene, average log2 TPM of each treatment is shown.

Plants dynamically synthesize specialized metabolites to protect themselves against biotic attack, one such class of metabolites are diterpenoids which have been identified as significant contributors to pest and pathogen resistance in maize (Block et al., 2019). In this study, we found several co-DEGs are involved in kauralexin and gibberellic acid (GA) biosynthesis pathways according to the result of KEGG pathways analysis (Supplementary Figure 1). To visually view expression changes of the genes involved in these two pathways, we selected the kauralexin and GA biosynthesis-related genes that had been reported previously, including maize *ent*-copalyl diphosphate synthase genes (*ZmAN1* and *ZmAN2*) (Schmelz et al., 2011), *ent*-kaurene synthase genes (*ZmKSL2*, *ZmKSL5*, and *ZmTPS1*), kaurene oxidase genes (*ZmKO1* and *ZmKO2*) and cytochrome P450 monooxygenase gene *ZmCYP71Z16* (Supplementary Table 2) (Ding et al., 2019). In maize, ZmAN1, ZmTPS1, ZmKSL5, and ZmKO1 are major enzymes involved in GA metabolism (Fu et al., 2016). In the present study, transcript levels of *ZmAN1, ZmTPS1, ZmKSL5*, and *ZmKO1* were down-regulated or unchanged after inoculation in all the groups (Figures 4A,B). Four kauralexin biosynthesis-related genes *ZmAN2*, *ZmKSL2*, *ZmCYP71Z16*, and *ZmKO2* were significantly up-regulated in all the seven inoculation groups (Figures 4A,B). These results suggested that maize GA metabolism was minimized and the kauralexin pathway was rapidly activated to protect maize against different pathogens invasion.

**FIGURE 5:**
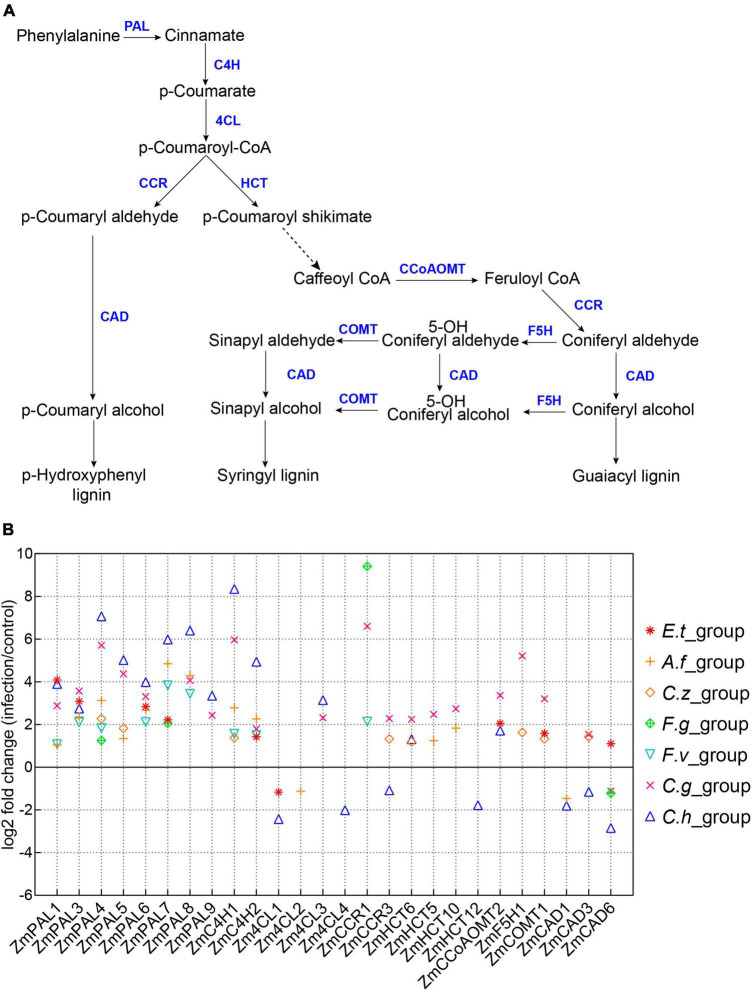
Expression patterns of lignin biosynthesis related genes. **(A)** A model of lignin biosynthesis. PAL, phenylalanine ammonia lyase; C4H, cinnamate 4-hydroxylase; 4CL, 4-coumarate CoA ligase; HCT, hydroxycinnamoyl CoA; CCR, cinnamoyl CoA reductase; CCoAOMT, caffeoyl CoA O-methyltransferase; F5H, ferulate 5-hydroxylase; COMT, caffeic acid O-methyltransferase; CAD, cinnamyl alcohol dehydrogenase. **(B)** Log2(Fold Change) of maize lignin biosynthesis-related genes, the seven inoculation groups are indicated by different symbols in the plot.

Lignin is a key component in plant secondary cell wall and plays a crucial role in plant innate immune defense system (Ma et al., 2018; Rossi et al., 2019). Accumulating evidences have suggested that rapid synthesis of lignin could effectively protect plants from pathogen invasion (Li C. et al., 2019). In this study, we also found a large number of DEGs involved in phenylpropanoid metabolism and lignin biosynthesis pathways (Supplementary Figure 2). We selected phenylpropanoid metabolism and lignin biosynthesis-related genes as reported previously to demonstrate the expression changes of these genes (Figure 5A and Supplementary Table 2) (Yang et al., 2017a). As expected, most phenylpropanoid metabolism and lignin biosynthesis-related genes expressions were up-regulated after inoculation with different pathogens (Figure 5B). We also observed several genes specific for lignin biosynthesis were down-regulated after pathogen infections, such as *ZmCAD6* and *Zm4CL1* (Figure 5B) (Rossi et al., 2019; Xiong et al., 2019).

### Nutrient transporter-related genes in maize in response to multiple pathogens invasion

Nutrient access is arguably the most limiting factor of pathogen invasion. Earlier studies of plant-pathogen interactions have identified a number of nutrient transporter genes (NTRs) participating in plant disease resistance. Based on recent studies (Chen et al., 2010; Moore et al., 2015; Sonawala et al., 2018; Eom et al., 2019), we chose two types of sugar transporter proteins and amino acid transporter proteins to demonstrate NTRs in maize response to multiple pathogens invasion, including maize SWEETs, STPs, and AATs. As illustrated in Supplementary Figure 3, more down-regulated DEGs were found in *SWEETs* after pathogens inoculation, with nine *SWEETs* being found to respond to at least two pathogens. Among those, *ZmSWEET2* (*Zm00001eb342040*), a gene related to sugar allocation in maize was significantly up-regulated by four pathogens (Sosso et al., 2015) (Supplementary Figure 3). By contrast, *STPs* and *AATs* showed more up-regulated DEGs after pathogens infection (Supplementary Figures 4, 5), with 33 *AATs* and 33 *STPs* being found to respond to at least two pathogens. Notably, an amino acid transporter gene (*Zm00001eb261480*) and two sugar transporter genes (*Zm00001eb098100* and *Zm00001eb377440*) were significantly up-regulated by all the seven selected pathogens in this study (Zhao et al., 2012).

### Identification and functional annotation of weighted gene co-expression network analysis modules associated with maize-pathogen interactions

**FIGURE 6:**
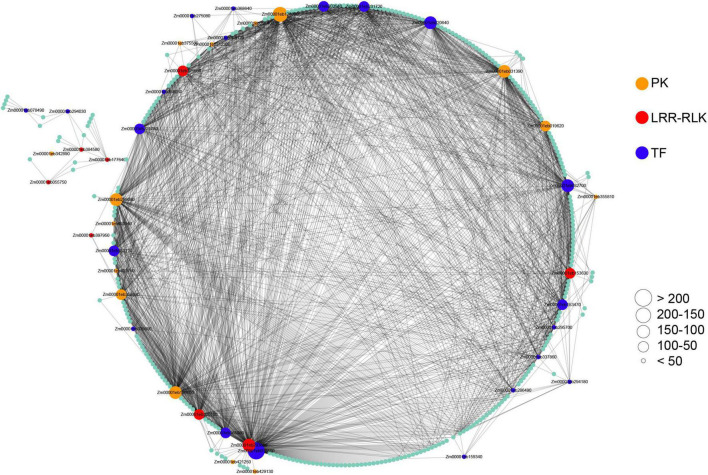
Co-expressed genes with edge weight >0.10 in module “salmon.” Each node represents a gene. Co-expressed genes are connected with a gray edge. Node size represents the number of co-expressed genes. PK, protein kinase; LRR-RLK, leucine-rich repeat receptor-like kinase; TF, transcription factor.

To identify maize-pathogen interactions associated modules and genes, we performed WGCNA using 25,646 expressed genes. The result of the module-trait relationships showed that module ‘salmon’ consisting of 516 genes is highly correlated with pathogens infection (Supplementary Figure 6, Supplementary Table 9), indicating that this module might play an important role in maize-pathogen interactions. To further explore this gene set, we focused on the genes encoding for transcription factors (TFs) and protein kinases (PKs), which might play key roles in the signaling network. For identification and visualization of TFs and PKs, the genes with WGCNA edge weight >0.10 were exported and visualized using Cytoscape. As a result, a total of 21 TFs and 23 PKs were identified, most of which were considered as hub genes of the network based on the number of interactions (Figure 6). Importantly, eight protein kinases were identified as LRR-RLKs. It is also interesting to note that six transcription factors were identified as WRKY TFs, including *WRKY64* (*Zm00001eb159340*), *WRKY8* (*Zm00001eb203940*), *WRKY114* (*Zm00001eb275080*), *WRKY83* (*Zm00001eb286490*), *WRKY82* (*Zm00001eb294180*), and *WRKY115* (*Zm00001eb368640*). Among these TFs, *WRKY82* and *WRKY115* have been reported to act as regulatory genes in the maize phenolic pathway (Yang et al., 2017a).

### Validation of representative common differentially expressed genes by quantitative RT-PCR

**FIGURE 7:**
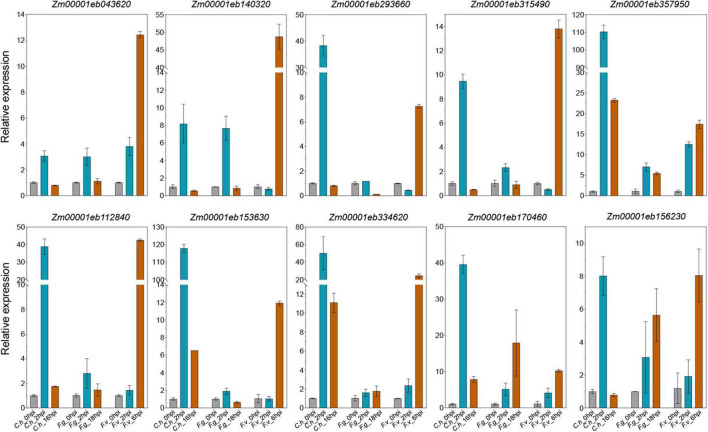
Quantitative reverse transcription-PCR (qRT-PCR) validation of representative co-DEGs. Data are represented as the mean ± SD from two biological repeats. *C.h, Cochliobolus heterostrophus*; *F.g, Fusarium graminearum*; *F.v, Fusarium verticillioides*.

We have identified a bunch of interesting co-DEGs that may be involved in multiple pathogens response. To validate the expression profiles from RNA-seq datasets, we inoculated maize inbred line B73 with *C. heterostrophus* on leaves, *F. graminearum* on roots, and *F. verticillioides* on ears, respectively. Ten representative co-DEGs were picked for qRT-PCR verification, including three *LRR-RLKs* (*Zm00001eb170460*, *Zm00001eb293660*, and *Zm00001eb153630*), two *WAK-RLKs* (*Zm00001eb334620* and *Zm00001eb156230*), one ATP binding cassette (ABC) transporter (*Zm00001eb357950*), one glutathione transferase 23 (*Zm00001eb315490*), one peroxidase (*Zm00001eb140320)*, one P450 (*Zm00001eb043620*), and one WRKY transcription factor (*Zm00001eb112840*). We found that most of the selected co-DEGs could be induced by all the three pathogens. All the 10 genes were significantly up-regulated by *C. heterostrophus* at 2 hpi and by *F. verticillioides* at 6hpi, while 9 of them were induced by *F. graminearum* at 2 hpi (Figure 7). The expression levels of the selected co-DEGs determined by qRT-PCR were in agreement with the changes in RNA-seq data except for *Zm00001eb293660*, whose expression was down-regulated by *F. graminearum* at 18 hpi (Figure 7), indicating that our analyses were accurate and reproducible. We hypothesize that the candidate genes identified here may be responsible for multiple disease resistance in maize.

## Discussion

Maize encounters different fungal pathogens throughout their lifetime. To cope with pathogen challenges, maize employs a variety of exquisite mechanisms to appropriately activate defense response. Until now, only a few genes have been identified and validated to confer disease resistance in maize (Yang et al., 2017b). Transcriptomics analysis of a susceptible plant-pathogen interaction can gain insights into both host defense responses and pathogen manipulation targets. In this study, we presented a meta-analysis of RNA-seq in maize induced by various fungal pathogens. We identified 115 co-DEGs in response to all the seven pathogens and 267 co-DEGs for the four pathogens infecting maize leaf. Diterpenoid and phenylpropanoid biosynthesis pathways were activated upon different pathogen invasions. Potential PRR, amino acid transporter, sugar transporter, transcription factor, and protein kinase genes that were induced by at least two pathogens were identified. This study highlights possible candidate genes that may be involved in multiple disease resistance in maize.

PAMP-triggered immunity (PTI) constitutes the first layer of plant immunity which has the potential to fend off diverse pathogens (Couto and Zipfel, 2016). Upon pathogen invasion, PTI was strongly activated, resulting in rapid and selective transcriptional reprogramming, induction of ROS, callose deposition, and production of hormones and antimicrobial compounds (Peng et al., 2018). In this study, we also found a considerable number of potential PRRs were highly induced by different pathogens, especially some *WAK-RLKs*. There are 7 *WAK-RLKs*, 4 *LRR-RLKs*, 3 *lectin-RLKs*, and 1 *LysM-RLK* were identified in the 267 co-DEGs (Supplementary Table 6). WAK-RLKs have been reported to confer quantitative disease resistance in different crops, such as maize (Hurni et al., 2015; Zuo et al., 2015), rice (Hu et al., 2017), and cotton (Wang et al., 2020). We identified a WAK-RLK gene *Zm00001eb360640*, which was induced by five pathogens selected in the study (Supplementary Table 8). This gene has been characterized by different groups which accounts for a major QTL for northern leaf blight resistance in maize (Hurni et al., 2015; Yang et al., 2021). There are 14 other *WAK-RLKs* showing enhanced expression by at least four pathogens, suggesting their probable roles in disease resistance. Very few *LRR-RLKs* have been reported to be involved in disease resistance in maize. In this study, 13 out of 115 identified LRR-RLKs were up-regulated by at least four pathogens. Two LRR-RLKs (*Zm00001eb293660* and *Zm00001eb153630*) identified as hub genes in the co-expression network module “salmon” were also included in the 267 co-DEGs (Figure 6). The mutants of *Zm00001eb293660* displayed enhanced susceptibility to *C. heterostrophus*, but increased resistance to *F. graminearum* (Block et al., 2021). The functions of these potential PRRs in maize disease resistance need to be further investigated in the future.

We also found 7 genes out of 267 (5 of 115) co-DEGs encode chitinase, two of which (*Zm00001eb167710* and *Zm00001eb167720*) locate on chromosome 3 as a cluster (Supplementary Table 6). *Zm00001eb167710* was also included in the co-expression network module “salmon,” which might be a key chitinase gene. Chitinases belong to four recognized families of pathogenesis-related (PR) proteins (PR-3, PR-4, PR-8, and PR-11) that have long been appreciated for their conserved role in the degradation of pathogen cell walls (Cletus et al., 2013; Sánchez-Vallet et al., 2015). Sixteen of the 267 (8 of 115) co-DEGs were cytochrome P450 genes, which are widespreadly involved in the diversification and functional modification of plant natural products, hormone regulation, plant defense, *etc.* (Liu et al., 2020; Pandian et al., 2020). Previous studies indicated that several P450 genes were involved in disease resistance in rice and barley (Ameen et al., 2021; Zhang et al., 2021; Wang A. et al., 2022), implying their potential roles in maize disease resistance. Interestingly, we also found that 8 of 267 (4 of 115) co-DEGs belong to the ABC transporter superfamily. The wheat gene *Lr34*, encoding an ABC transporter, confers partial resistance to multiple fungal diseases (Krattinger et al., 2009). Arabidopsis *PEN3/PDR8* encoding an ABC transporter has also been reported to be involved in disease resistance (Stein et al., 2006). In addition, two AATs and three STPs were identified in the co-DEGs (Supplementary Table 6). It would be interesting to determine the function of these genes in different disease resistance in maize in the future.

The secondary metabolic process was one of the most highly enriched GO terms among the DEGs. The production of antimicrobial compounds are critical components of plant immunity. Earlier studies have found kauralexins as significant contributors to protect against fungal pathogens in maize (Ding et al., 2019). Upon pathogen invasion, maize kauralexin pathway could be rapidly activated to enable the biosynthesis of *ent*-kaurene-related antibiotics, while at the same time the production of GA metabolic precursors were minimized to avoid dysregulated phytohormone signaling induced by pathogens (Lu et al., 2015). Our meta-analysis showed *ent*-kaurene synthesis-related genes were commonly up-regulated in seven inoculation groups (Figure 4B). We speculate that kauralexin pathway may positively respond to multiple pathogens in maize.

The plant cell wall is a dynamic barrier that many pathogens will first encounter. Lignin is one of the main components of plant cell wall biopolymer. Phenylpropanoid biosynthesis pathway was highly enriched in our analysis (Figure 3C). Maize genes encoding gateway enzymes of the phenylpropanoid pathway such as phenylalanine ammonialyases (PALs) and cinnamate-4-hydroxylases (C4Hs) (Dong and Lin, 2021) were highly induced by most pathogens (Figure 5B). But for the downstream enzymes of phenylpropanoid pathway, some of the genes were not significantly induced or even down-regulated by pathogens, such as *Zm4CL1*. Mutation of this gene affects lignin synthesis and increases the cell wall digestibility, implying that over-expression of *Zm4CL1* might enhance maize disease resistance (Xiong et al., 2019). Among those genes, *ZmCCoAOMT2* and *ZmCAD* have been reported to confer quantitative disease resistance to southern leaf blight, gray leaf spot, and banded leaf and sheath blight in maize, respectively (Yang et al., 2017c; Li N. et al., 2019). Knockdown of *Hydroxycinnamoyl-CoA shikimate/quinate hydroxycinnamoyl transferases* (*HCTs*) led to the redirection of metabolic flux to the biosynthesis of flavonoids (Hoffmann et al., 2004). These results suggest that phenylpropanoid metabolism and lignin biosynthesis-related genes might be good targets for genetic engineering to improve broad-spectrum disease resistance in maize.

Phytohormones play important roles in plant disease resistance. The biosynthesis and metabolism of salicylic acid (SA) and jasmonic acid (JA) have profound importance in plant immunity (Borrego and Kolomiets, 2016; Zhang and Li, 2019). Increasing evidence indicates that SA is well-integrated into both PTI and ETI (Zhou and Zhang, 2020). We also found maize SA marker genes *ZmPR1* (*Zm00001eb299370*) and *ZmPR5* (*Zm00001eb032600*) were highly induced by different pathogens (Supplementary Table 6). Interestingly, several other *PR5* genes that have been annotated as thaumatin-like protein genes were identified in the co-DEGs. Plant immunity requires large-scale transcriptional reprogramming, where TFs and PKs play critical roles. Our co-expression network analysis showed that some TFs and PKs were closely related to pathogen induction. More importantly, most of these genes act as the hubs of the network (Figure 6). A homeobox transcription factor (Zm00001eb015500) displayed close interaction with many hub genes. Recent studies suggest that TFs and PKs are key regulators in coordinating yield and immunity (Wang et al., 2018; Wu et al., 2020). These genes may be putative candidates for maize disease resistance.

In conclusion, our analysis revealed the importance of phenylpropanoid and diterpenoid biosynthesis pathways in maize disease resistance. Further understanding of the identified candidate genes including *PRRs*, *NTRs*, *TFs*, and *PKs*, will facilitate disease resistance breeding in maize.

## Data availability statement

The datasets presented in this study can be found in online repositories. The names of the repository/repositories and accession number(s) can be found in the article/Supplementary material.

## Author contributions

QY and YW conceived and designed the research. YW and TL analyzed the data and wrote the manuscript. HT, XH, NY, and ZS revised the manuscript. QY led the project and revised the manuscript. All authors have read and approved the final manuscript.

## Footnotes

1
https://www.ebi.ac.uk/Tools/hmmer/

2
http://kobas.cbi.pku.edu.cn/kobas3/

3
http://planttfdb.gao-lab.org/

4
https://www.maizegdb.org/
