# Supplementary material for: Comparative transcriptome meta-analysis reveals a set of genes involved in the responses to multiple pathogens in maize
Source: Front Plant Sci. 2022 Sep 15;13:971371. doi: 10.3389/fpls.2022.971371 (PMC9521429; doi:10.3389/fpls.2022.971371)
Supplement: Supplementary file 2 [file Table_2.docx]

**Supplementary Materials**

**Supplementary Table 1.** List of transcriptomic libraries analyzed in this study.

**Supplementary Table 2.** List of kauralexin and lignin biosynthesis-related genes

**Supplementary Table 3.** Primers used for qRT-PCR analysis in this study.

**Supplementary Table 4.** The transcripts per million matrix of 25646 filtered genes.

**Supplementary Table 5.** Differentially expressed genes in this study.

**Supplementary Table 6.** List of common differentially expressed genes (co-DEGs).

**Supplementary Table 7.** Gene ontology enrichment analysis of differentially expressed genes. Genes were annotated in three main categories: biological process (BP), cellular component (CC), and molecular function (MF).

**Supplementary Table 8.** Potential pattern recognition receptors (PRRs) identified in the study. Values are presented as log2fold change of each gene in different inoculation group.

**Supplementary Table 9.** List of 516 genes in the module ‘salmon’. TFs and PKs are highlighted in yellow.

**Supplementary Figure 1.** The differentially expressed genes (DEGs) involved in diterpenoid biosynthesis. Red boxes indicate DEGs, green boxes indicate organism-specific genes.

**Supplementary Figure 2.** The differentially expressed genes (DEGs) involved in phenylpropanoid biosynthesis. Red boxes indicate DEGs, green boxes indicate organism-specific genes.

**Supplementary Figure 3.** The differentially expressed maize sweet transporter genes. Values are presented as log2 fold-changes between control and pathogen treatment in each group.

**Supplementary Figure 4.** The differentially expressed maize sugar transporter genes.

Values are presented as log2 fold-changes between control and pathogen treatment in each group.

**Supplementary Figure 5.** The differentially expressed maize amino acid transporter genes. Values are presented as log2 fold-changes between control and pathogen treatment in each group.

**Supplementary Figure 6.** Co-expression network constructed by weighted gene co-expression network analysis. (A) Hierarchical clustering dendrogram used for 34 RNA-seq data. (B) visualization of the soft-threshold value. (C) Module-trait relationships. Each row corresponds to a module eigengene, column corresponds to a trait. Each cell contains the corresponding correlation (first line) and *p*-value (second line). (D) Eigengene expression profile of module ‘salmon’. A high value means a lot of module genes are over-expressed (red in the heatmap) and a low value means under-expressed (green color in the heatmap).
