# Supplementary figures and images for: Comparative transcriptome meta-analysis reveals a set of genes involved in the responses to multiple pathogens in maize
Source: Front Plant Sci. 2022 Sep 15;13:971371. doi: 10.3389/fpls.2022.971371 (PMC9521429; doi:10.3389/fpls.2022.971371)

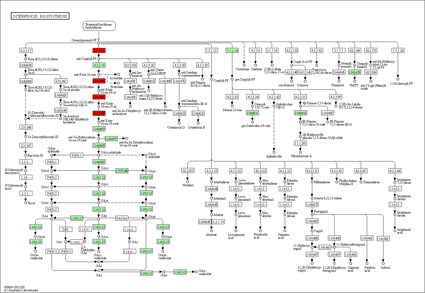

Supplement: Supplementary file 3 [file Image_1.png]

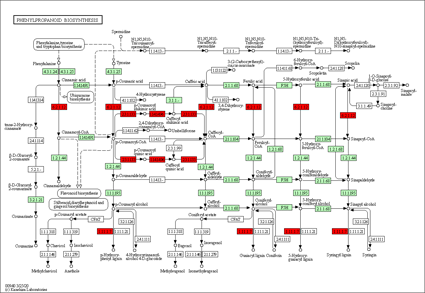

Supplement: Supplementary file 4 [file Image_2.png]

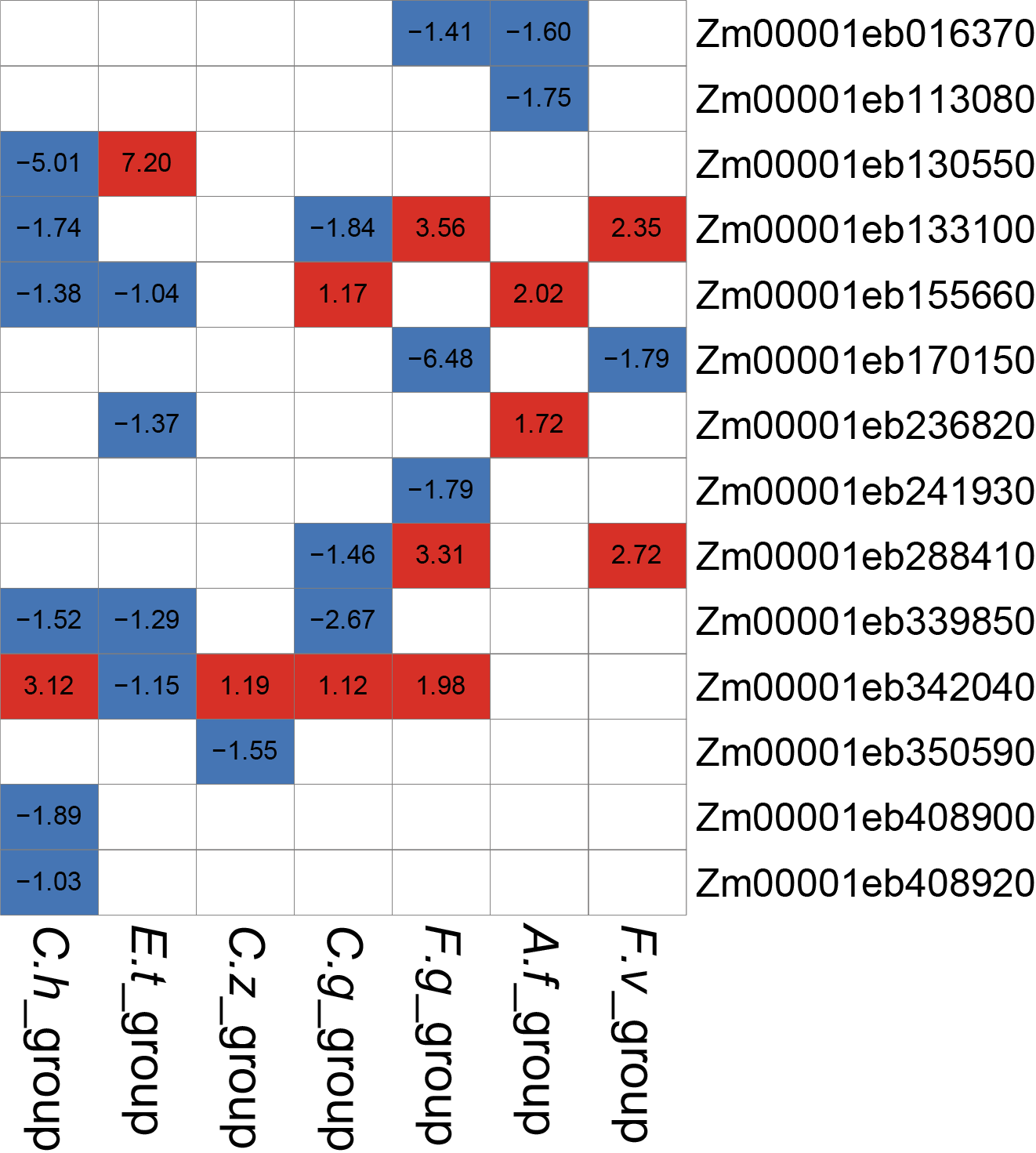

Supplement: Supplementary file 5 [file Image_3.tif]

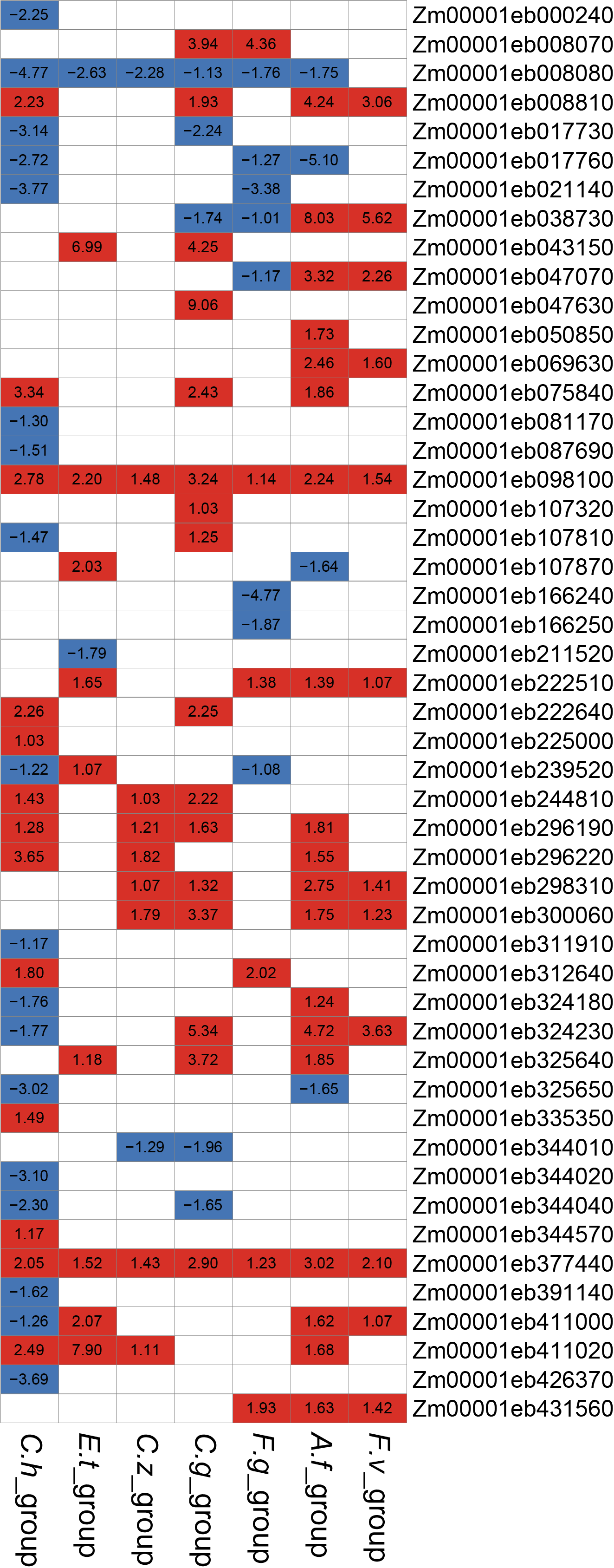

Supplement: Supplementary file 6 [file Image_4.tif]

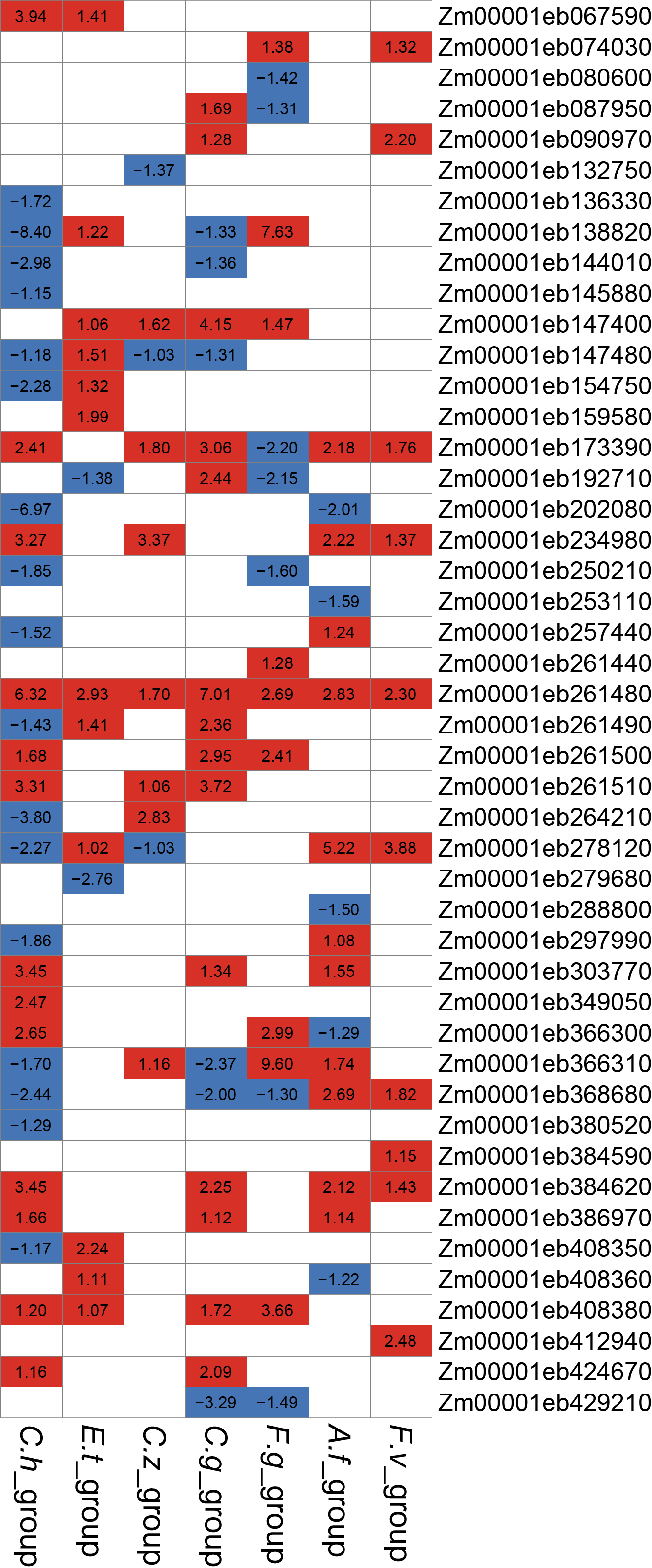

Supplement: Supplementary file 7 [file Image_5.tif]

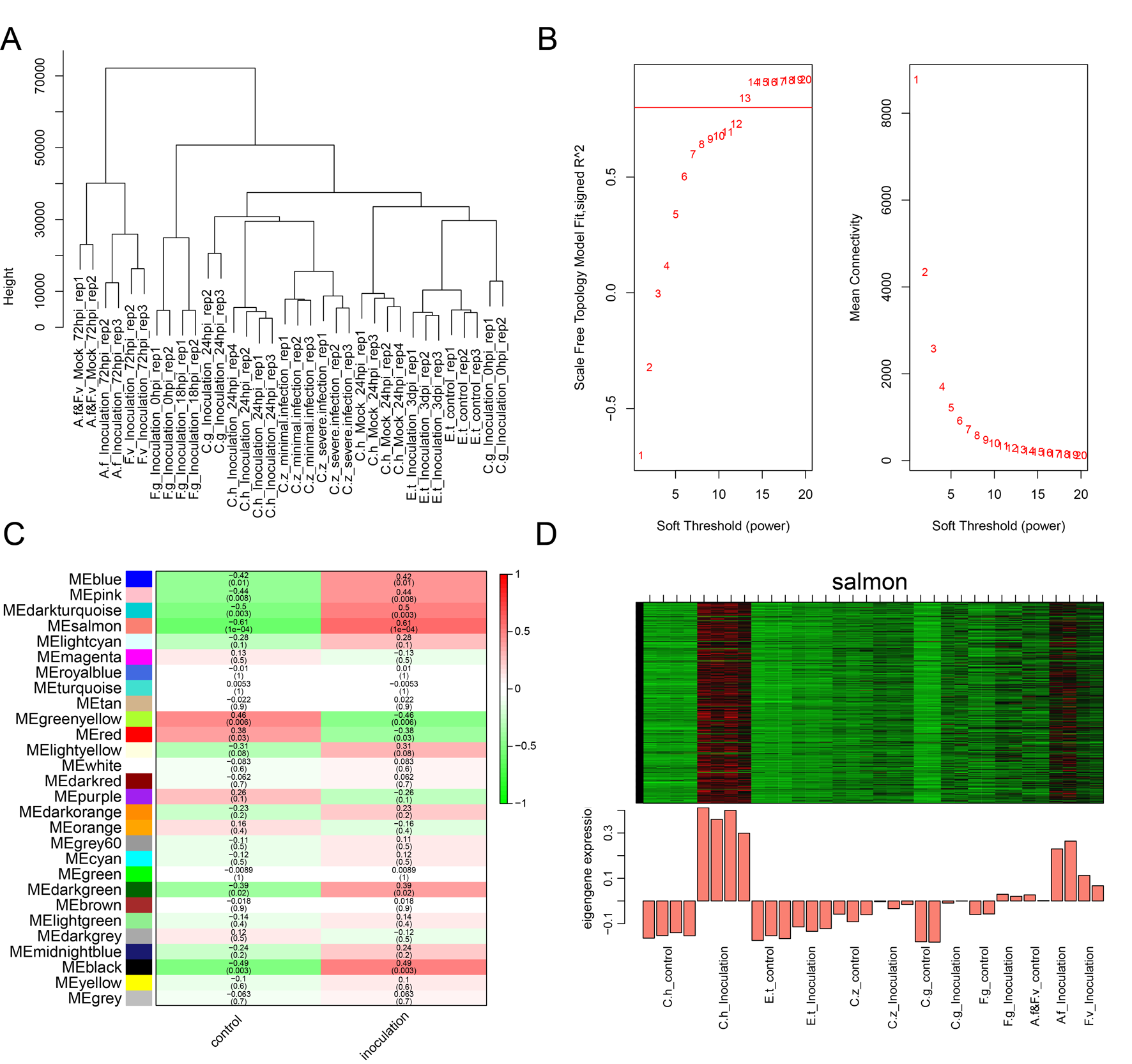

Supplement: Supplementary file 8 [file Image_6.tif]
